# Overview of surgical training and assessment of surgical skills: a narrative review

**DOI:** 10.3389/fsurg.2025.1605495

**Published:** 2025-06-25

**Authors:** Margareta Berg, Lars B. Dahlin, Magnus Kjellman

**Affiliations:** ^1^ Department of Neurosurgery, Sahlgrenska University Hospital, Gothenburg, Sweden; ^2^ Department of Translational Medicine—Hand Surgery, Lund University Sweden, Lund, Sweden; ^3^ Department of Molecular Medicine and Surgery, Karolinska Institutet, Stockholm, Sweden

**Keywords:** surgical training, review of reviews, surgical skills, assessment of surgical skills, evidence-based training and assessment methods

## Abstract

**Objectives:**

To assess the existence of international, scientifically validated methods for manual surgical skills training and assessment. The aim was to create a descriptive chronological summary of the existing evidence and conclusions in the included articles.

**Methods:**

PubMed and Scopus were searched twice for reviews published between 1997 and October 2023 and between 1997 and January 2024. The search terms used were “review,” “surgical training,” “surgical skills,” “assessment,” and “evaluation” in combinations. In all, 38 reviews were included (systematic and non-systematic). In addition, 30 non-reviews were selected for the introduction and the historical background. Publications on non-technical skills were excluded.

**Results:**

Great efforts have been invested by committees and working groups to define methods for surgical training and assessment. However, the work was found to be scattered, without attempts to be overarching and internationally valuable, and few training methods were strictly scientifically validated. Many reviews were limited to (1) one surgical procedure, (2) to one surgical specialty, (3) to one surgical method such as endoscopic procedures or “robotics,” or (4) to a limited geographical area.

**Conclusions:**

Scientifically validated, generally applicable methods for surgical training and assessment could not be found. Further research is needed to find simple, equal, and overarching methods to allow valid and reliable comparisons of manual surgical competencies across borders, in financially strained healthcare where resources for adequate training and evaluation of skills may not be available. Financial aspects can be included in future studies, to correlate costs for training with costs for avoidable surgical complications.

## Introduction

This study aimed to investigate whether there are published, rigorously scientifically validated methods for surgical education and assessment that are evidence-based and that can be applied generally across countries and all types of surgery. We presumed that a certain level of basic education is required before specialization.

Human anatomy is essentially the same, regardless of country, culture, religion, and socioeconomic status. For this reason, the surgical profession should be equal and comparable between countries and continents. This topic includes the associated questions of surgical training and assessment, surgical errors, and patient safety, as well as acknowledging that surgeons are humans making mistakes (1). Anyone of us might end up on an operation table tomorrow. The question of surgical safety is regarded as closely related to the UN Declaration of Human Rights ratified on 16 September 1949, which in this context affirms that each individual has the right to be operated on by a properly trained surgeon when a surgical procedure is required. There is scientific evidence that surgical treatment includes risks for errors, injuries, and deaths (1–4), but it is believed that validated statistics on healthcare injuries are scarce and seldom systematically divided into surgical or non-surgical (5). In a 2023 scoping review on surgical informed consent (6), surgical risk factors and outcomes were discussed, excluding the impact of the surgical intervention itself.

For the last 30 years, there have been an increasing number of publications on “surgical training” and “assessment of surgical skills,” but many of these studies lack consistency and often contain a mix of other competencies—such as diagnostics, judgment of indication for surgical interventions, mentoring, work-based assessment, and other non-technical skills such as leadership, teamwork, and work–life balance (7–22). Virtual reality training, other forms of simulation, and tools for surgical training have been widely described, most often in endoscopic surgery (23–31), while studies on simulation of open surgery, for example, cadaver training, are rare (32, 33). Complicating factors, such as the use of rapidly developing technical surgical equipment, have raised the issue of manual skills, training, and certification processes even in other scientific domains such as educational and technical sciences as well as human factors (34–37). A 2008 report on “deliberate practice” (34) from educational sciences gained substantial interest (7), as it showed that it would take 10 years or 10,000 h of deliberate practice for someone to demonstrate manual expertise. Not only the *method* of training but also the *structure* and *transfer* of the practice from the training situation to the OR were regarded as equally important by the author and mentioned in some of the included reviews (38–42).

A large number of surgical associations and different working groups (Table 1) have paid immense efforts to find solutions for surgical training methods (Table 2) that rarely seem to be subsequently validated in scientific studies.

**Table 1:** Examples of surgical organizations, working groups, and societies working with surgical training methods mentioned in the included reviews.

Organization	Country	Committees	Surgical specialty	Included reference
American Board of Surgeons (ABS)	USA		All	Buschemeyer et al. (52)
American College of Surgeons (ACS)	USA	Safety and Efficacy of Innovative Procedures and Technology in Surgery	All	Buschemeyer et al. (52)
Accreditation Council for Graduate Medical Education (ACGME)	USA, combined from American Board of Medical Specialities; Association of American Medical Colleges; Council on Medical Specialty Societies; American Medical Association; American Hospital Association	Outcomes Project: Core Competencies and Milestones Milestone Working Group Resident Review Committee for Surgery (RRC-S) Institutional Review Committee (IRC) Next Accreditation System (NAS) Clinical Competency Committees (CCCs)	All	Buschemeyer et al. (52)
ACCME	USA	The Accreditation Council for Continuing Medical Education		Buschemeyer et al. (52)
American Society of Breast Surgeons (ASBS)	USA			Buschemeyer et al. (52)
Surgical Council of Resident Education	USA		All	Buschemeyer et al. (52)
Association for Surgical Education (ASE)	USA		All	Buschemeyer et al. (52)
Association of Program Directors in Surgery (APDS)	USA		All	Buschemeyer et al. (52)
American Board of Medical Specialties (ABMS)	USA		All	Buschemeyer et al. (52)
Centre for Advanced Surgical Technologies (CAST)	Louisville, USA		All	Buschemeyer et al. (52)
Society of American Gastrointestinal and Endoscopic Surgeons (SAGES)	USA		Endoscopic surgery	Buschemeyer et al. (52)
Association of Professors of Gynecology and Obstetrics Undergraduate Medical Education Committee	USA		Obstetrics and gynecology	Hammoud et al. (53)
The Royal College of Physicians and Surgeons of Canada	UK		All	Schaverien (7)
Royal College of Surgeons (RCS)	UK		All	Gough (2011)
Royal College of Surgeons of Ireland (RCSI)	Ireland		All	Gough (2011)
Royal College of Edinburgh (RCE)	Scotland		All	Gough (2011)
Royal Australasian College of Surgeons (RACS)	Australia, New Zealand, Asia		All	Gough (2011)
Royal Australasian College of Surgeons Trainees Association (RACSTA)	Australia, New Zealand, Asia		All	Gough et al. (2011)
Australian Safety and Efficacy Register of New Interventional Procedures-Surgical (ASERNIP-S)	Australia, New Zealand, Asia	Royal Australasian College of Surgeons	All	Gough et al. (2011)
General Medical Council (GMC)	UK		All	Shalhoub et al. (9)
MMC	UK	Modernising Medical Careers	All	Shalhoub et al. (9)
Association of American Medical Colleges, Institute for Improving Medical Education	USA	ADDIE Framework	All	Sanfey (10)
SIU	USA	Southern Illinois University	General surgery	Sanfey, (10)
JCST	UK	Joint Committee on Surgical Training	All	Shalhoub et al. (9), Humm et al. (64)
BOTA	UK	British Orthopaedic Trainees Association	Orthopedic surgery	Shalhoub et al. (9)
HQIP	UK	Healthcare Quality Improvement Partnership, NIH	All	Radford et al. (56)
IRCAD	France, Taiwan, Brazil	L'Institut de Recherche contre les Cancers de l’ Appareil Digestif	Gastrointestinal	Forgione et al. (2022)
AIMS	Italy	Academy for International Minimally Invasive Surgery	Minimally invasive	Forgione et al. (2022)
MATTU Centre	UK	Minimal Access Therapy Training Unit	Minimally invasive	Forgione et al. (2022)
Cushieri's Skill's Centre	Scotland	Dundee University	All	Forgione et al. (2022)
MITIE	USA	Methodist Institute for Technology, Innovation and Education	All	Forgione et al. (2022)
Centre for the Future of Surgery	USA	San Diego	All	Forgione et al. (2022)
European Surgical Institute	Germany	Norderstedt	All	Forgione et al. (2022)
European Association for Endoscopic Surgeons (EAES)				Forgione et al. (2022)
American Society for Gastrointestinal Endoscopy (ASGE)	USA			Forgione et al. (2022)

**Table 2:** Examples of acronyms or names for surgical training and assessment methodologies found in the included reviews.

Acronym	Definition	Included reference
OSATS	Objective structured assessment of technical skill system	Martin et al. (45), Hammoud et al. (53), Sanfey et al. (2014), Forgione et al. (51), Fritz et al. (16)
CME	Continuous medical education	Buschemeyer et al. (52)
SBT	Simulation-based training	Hammoud et al. (53)
ISCP	The intercollegiate surgical curriculum program, UK	Schaverien (7)
SET	Surgical education and training program	Gough (2011)
VR	Virtual realty	Zevin et al. (11)
ICSAD	Imperial college surgical assessment device, UK	Sanfey et al. (2014)
WBA	Workplace-based assessments	Shalhoub et al. (9), Aryal et al. (49)
PBA	Procedure-based assessment	Shalhoub et al. (9), Mayne et al. (62), Aryal et al. (49)
DOPS	Direct observation of procedural skill	Shalhoub et al. (9), Mayne et al. (62)
CTA	Cognitive task analysis	Sanfey et al. (2014)
GOALS	Global operative assessment of laparoscopic skills	Sanfey et al. (2014), Henning et al. (66)
STEPP	Status of the patient, team, environment, and progress toward the goal	Sanfey et al. (2014)
VOP	Verification of proficiency system (SIU, Table 1)	Sanfey et al. (2014)
SSD	Surgeon-specific outcome data	Radford et al. (56)
MISTELS	McGill inanimate system for training and evaluation of laparoscopic skills	Sanfey et al. (2014), Fritz et al. (16)
ICSAD	Imperial college surgical assessment device	Sanfey et al. (2014), Fritz et al. (16)
OPRS	Operative performance rating system, ACGME	Sanfey et al. (2014), Fritz et al. (16)
SMART	Situation, management, activity, rapidity, troubleshoot	Sanfey (10)
AES	Assigned educational supervisor	Shalhoub et al. (9)
AoP	Assessment of practice	Shalhoub et al. (9)
ARCP	Annual review of competency progression	Shalhoub et al. (9)
CBD	Case-based discussion	Shalhoub et al. (9)
CCT	Certificate of completion of training	Shalhoub et al. (9)
CT	Core training	Shalhoub et al. (9)
Miller's pyramid	Assessing clinical competence by grading in four steps from novice to expert: “knows, knows how, shows, does”	Shalhoub et al. (9), Aryal et al. (49)
Mini-CEX	Mini-clinical evaluation exercise	Shalhoub et al. (9)
Mini-PAT	Mini-peer assessment tool	Shalhoub et al. (9)
MSF	Multisource feedback	Shalhoub et al. (9)
OOT	Observation of teaching	Shalhoub et al. (9)
PROMs	Patient-related outcome measures	Shalhoub et al. (9)
SLE	Supervised learning event	Shalhoub et al. (9)
ST	Specialty training	Shalhoub et al. (9)
TAiP	Training and assessment in practice	Shalhoub et al. (9)
TOOR	Training outside the operating room	Forgione et al. (51), Freischlag (2021), Oral Communication Third Surgicon Congress
MOOC	Massive open online course	Evans et al. (43)
SCORE	Surgical council on resident education	Evans et al. (43)
WISE-MD	Web initiative for surgical education of medical doctors	Evans et al. (43)
WebSurg	Virtual surgical university, accessible through the Internet	Evans et al. (43)
SICKO	Surgical improvement of clinical knowledge ops	Evans et al. (43)
Kirkpatrick's model	Four-level model for evaluating training programs	Maertens et al. (39)
GRADE	Grading of recommendations assessment, development, and evaluation	Maertens et al. (39)
RCT	Randomized controlled trials	Maertens et al. (39)
CONSORT	Checklist for randomized controlled trials	Maertens et al. (39)
NOTSSdk	Non-technical skills for surgeons in Denmark	McKendy et al. (58)
SHARP	**S**et learning objectives, **H**ow did it go?, **A**ddress concerns, **R**eview learning points, and **P**lan ahead	McKendy et al. (58)
BID	Briefing, intraoperative, debriefing	McKendy et al. (58)
O-SCORE	Ottawa surgical competency operating room evaluation	Fritz et al. (16)
OPRS	Operative rating system	Sanfey (10), Fritz et al. (16)
Zwisch scale	Analysis of intraoperative behavior, using four stages of supervision: show and tell, smart help, dumb help, and no help	Fritz et al. (16)
CBE	Competency-based education	Ashmore et al. (15)
PICO	Population, intervention, comparison, outcome	Williams et al. (63)
Likert scale	Usually a 5- or 7-point ordinal scale used by respondents to rate the degree to which they agree or disagree with a statement	Williams et al. (63)
PBA	Procedure-based assessment	Mayne et al. (62), Aryal et al. (49)
SAVE	Simulation-based trauma scenarios of increasing difficulty: surgery for abdomino-thoracic violence	Riaz et al. (2020)
WBA	Workplace-based assessment	Aryal et al. (49)
CCT	Certificate of completion of training in general surgery	Humm et al. (64)
VSSI	Vaginal surgical skills index	Henning et al. (66)
HASC	Hopkins assessment of surgical competency	Henning et al. (66)
OSALS	Objective structured assessment of laparoscopic salpingectomy	Henning et al. (66)
RHAS	Robot hysterectomy assessment score	Henning et al. (66)
CAT-LSH	Competence assessment for laparoscopic supracervical hysterectomy	Henning et al. (66)
“Feasible rating scale for formative and summative feedback”	Blinded video assessment by two observers	Henning et al. (66)
GRS	Global rating scale	Henning et al. (66)
iVR	Immersive virtual reality	Mao et al. (28)
MCQ	Multiple-choice question	Han et al. (46)
SAGES Online	Online educational training site	Forgione et al. (2022)
COSATS, GOSATS	*Colorectal* or *gynecological* surgery: objective structured assessment of technical skill system	Han et al. (46)
FLS	Fundamentals of laparoscopic surgery	Zevin et al. (11), Han et al. (46)
FES	Fundamentals of endoscopic surgery	Han et al. (46)
GAGES	Global assessment of gastrointestinal endoscopic skills	Han et al. (46)
ABSITE	Am board of surgery in training examination	Han et al. (46)
QE	Qualifying examination	Han et al. (46)
CE	Certifying examination	Han et al. (46)
GERT	Generic error rating tool	Henning et al. (66), Han et al. (46)
STSF	Structured technical skills assessment form	Hurriez et al. (2019)
TBC	Task-based checklist	James et al. (32)
GRS	Global rating scale	James et al. (32)
FPA	Final product analysis	James et al. (32)
MERSQI	Medical education research quality index	James et al. (32), Mao et al. (28)
ASSET	Arthroscopic surgical skill evaluation tool	James et al. (41)
CBE	Competency-based education	Stahl et al. (21)
EPA	Entrustable professional activities	Stahl et al. (21)
GPC	Generic professional capabilities	Aryal et al. (49)
C-SATS	Crowd-sourced assessment of technical skills	Cardoso et al. (68)

From technical sciences (37), a study of the complexity of telemedicine and human factors tried to propose new ways of thinking forward in the conclusion: “According to numerous reviews and policy documents, system dynamics and complexity should be considered during the design and evaluation of technological change in health care,” and “this analysis serves as an example of how a complex clinical implementation context can be analysed and represented in a granular yet structured manner while also showing the interactions among the system elements.” In a 2016 study on general surgery in the USA (43), it was stated that “Simulation, VR, robotics, telemedicine, and gaming are no longer the future of surgical education, but represent the standard by which competence will be developed and measured.” In an interdisciplinary dissertation from the educational sciences and orthopedic surgery in 2022 (44), it was stated that “there is a lack of consensus among surgical educators or research into the required core skills and evaluation of their performance.” In this work, the Delphi method was used “to provide baseline data for future studies to reach a meaningful consensus to designing effective surgical training and evaluation of core surgical techniques.”

**Figure 1:**
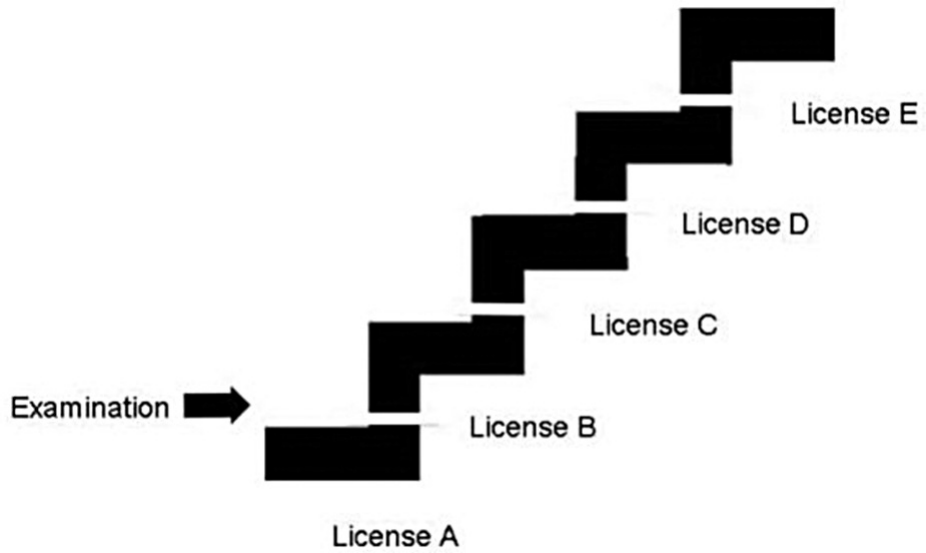
The surgical staircase model with interruptions at each level symbolizing practical examinations (47).

Examples of studies showing scientifically validated surgical training and assessment *methods* are available, such as the Objective Structured Assessment of Technical Skills (OSATS) system (45) since 1997, methods described in VR training in laparoscopic cholecystectomies in 2002 (24), and the proficiency-based progression training curriculum for an isolated endoscopic surgical procedure in 2015 (13) (arthroscopic Bankart surgery). In the latter, methods for objective measurements of manual skills were described, comparing the video of a performed surgical procedure to a standard video of the same surgical procedure. The standard video was fragmented into a number of mandatory elements to perform a correct arthroscopic Bankart procedure, and from the actual real-life video, the number of fulfilled elements was counted, in this way presenting a way to measure the surgical result. In a 2021 study on general surgery in the USA (46), “the need for standardized surgical training in this era of evidence-based medicine” was emphasized, mainly referring to mandatory multiple-choice questionnaires as an assessment method. However, it was also stated that written examination elements of board certifications did not correlate to the readiness to practice surgery, and assessment methods such as COSATS and GOSATS (Table 2) were validated but “not to be implemented as a requirement for board certification due to the costs and efforts needed to execute such assessments of surgical skills.” The question was raised “whether it is more appropriate to relegate assessment of technical competence to standardized examinations such as these or to the judgment of program directors who have access to more extensive, longitudinal evaluations of trainee performance in this domain.” A hypothesis of a generally applicable *structure*, with a stepwise training staircase model, was presented in 2017 (47, 48) (Figure 1), where it was presumed that surgical associations for one selected specialty might agree on the content of Level 1, Level 2, Level 3, etc., even between different countries. It was suggested that no time limit should be included for the acquirement of skills, but instead the passing of a defined clinical examination of some type to be allowed to move from one level to the next, as the time of the learning curve might vary between individuals. It was concluded that such a hypothesis of a staircase structure needs to be tested by high-quality scientific research such as randomized controlled trials (RCTs) to be supported or rejected.

More recent work suggests the use of “work-base assessment” as a standardized tool for evaluation of surgical skills (49). A 2023 scoping review on surgical outcomes excluded the very surgical intervention as a factor influencing surgical outcomes (50). In our review, focus is paid only to the aspects of manual surgical skills, and non-technical skills are excluded. Our focus is only on training in surgical skills and assessment methods of such skills, both for beginners and mature surgeons learning new surgical techniques or instruments.

## Methods

In order to find any available information on scientifically validated, internationally accepted, and generally applicable methods for training and assessment of surgical skills, regardless of surgical specialty, a literature search was made only for scientific reviews by one professional librarian and two reviewers in October 2023. The same search was remade in January 2024. PubMed and Scopus were searched for relevant reviews published from 1997 to October 2023 and then from 1997 to January 2024. The search terms used were *review*, *surgical training*, *surgical skills*, *assessment*, and *evaluation* using boolean operators as *and* and *or*. Both searches were limited to titles only.

### Identifying relevant studies

**Figure 2:**
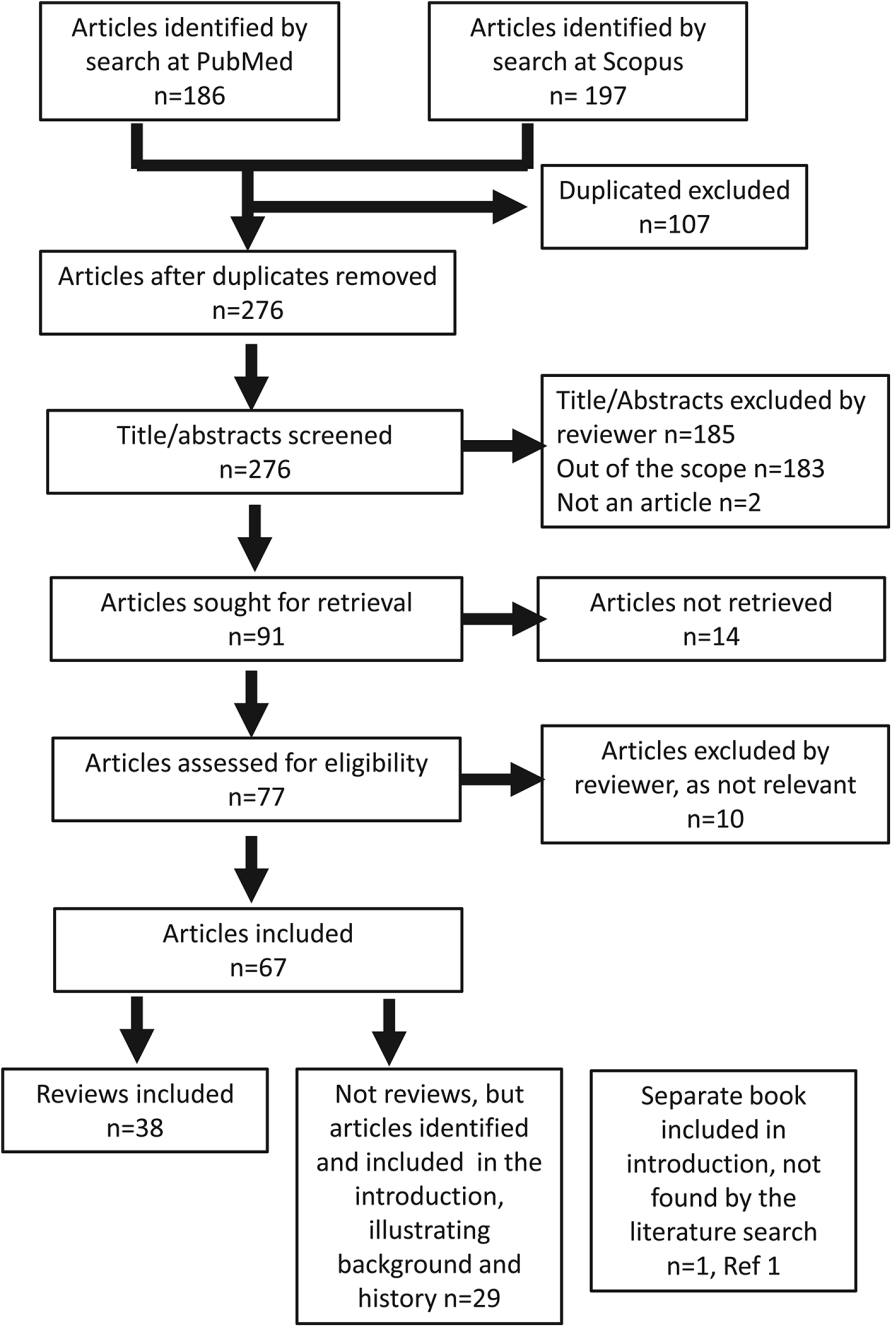
Prisma flow diagram, a summary of two searches.

The literature search resulted in 383 articles. After removing 107 duplicates, 276 articles were screened by title and abstract, of which 185 articles were excluded, including those out of the scope (183) or not being actual articles (2). A total of 91 articles were sought for retrieval in open-access articles, and 14 were not retrieved. In addition, 77 articles were accessed for eligibility, and 10 were excluded by the reviewers as they were not relevant. After full-text screening, a total of 38 reviews were included. In addition, 29 non-review articles were included in the introduction to illustrate the background of the topic (Figure 2). One additional book was included in the reference list, used in the introduction.

## Results

### Summarizing the included reviews

Of the included 38 reviews, 14 were found to be generally applicable, while 16 reviews were limited to one specific surgical procedure, to one diagnosis such as “kidney cancer,” to a special area such as “robotics” or “laparoscopy,” to one surgical specialty such as “urology,” or to a specified geographical region. However, conclusions were strikingly similar, often stating that the actual study was limited and further research would be needed to draw general conclusions. Only two of the reviews mentioned the *international* aspect of surgical training and assessment (8, 51).

### Collating and reporting the results

The search outcome is given as a descriptive chronological overview of the reviews and their conclusions, to illustrate the history, development, scientific quality, the number of involved factors, and the status of the topic.

### Results of the search

In a 2005 study on general surgery (52), it was stated that “surgery necessarily entails patient morbidity and mortality” and that “the rapid technological development is changing the surgical profession.” The challenge of defining the processes of accreditation, certification, credentialing, and privileging was pointed out, and the suggested definitions are “blurred, blended, occasionally misrepresented and misunderstood.” It was also emphasized that laparotomy and laparoscopy “now should be learned in the same amount of time” as open surgery used to be learned as the exclusive approach. Training after residency was found to lack uniform structure, organization, detailed guidelines, and nationally recognized criteria. Animal laboratories and mechanical simulators were described as excellent ways of training outside the operating room (TOOR) to simulate surgery and shorten the learning curve, but such training was seen as labor-intensive and expensive. In an attempt to standardize surgical training, a Centre for Advanced Surgical Technologies (CAST) was created at the University of Louisville, where approximately 20 predefined surgical procedures in general surgery were systematically trained in a rotation program during 5 years in residency. One conclusion was: “The surgical community is the most knowledgeable and appropriate group to issue training guidelines.”

A committee from obstetrics and gynecology (53) stated in 2008 that simulation-based surgical training was used with limited evidence-based data to support the validity and reliability. When discussing low- and high-fidelity simulators, it was concluded that: “…a more robust assessment program for surgical skills potentially can be achieved with a combination of OSATS, performance assessments from virtual reality simulators, and global rating scales from observation of a trainee operating on a real patient.”

In the UK in 2011, the general value of cadaver workshops (54) was “held in high regard and felt they help to improve operative skills” but showed that validated data on real-life skill's *transfer* from cadaver workshops to the operating room are missing in the literature and emphasized the need for further scientific studies.

Hand surgeons emphasized in 2011 the need for formalized programs for surgical mentorship (38) and stated that the surgical environment is unique and defined by several distinct characteristics that set it apart from other professional settings in health care: “the expectations combined with the stress associated with the operating room, present a challenging learning environment for surgical trainees of all disciplines.”

International aspects of the use of the Internet and modern communication technology in surgical training were considered in obstetrics and gynecology from Sweden and Spain in 2012 (8). Telementoring, teleproctoring, and robotic telesurgery were judged to have great potential, and it was concluded that: “The integration between surgery and telecommunications could constitute one of the major achievements of modern medicine, and its safe integration into clinical practice should be a priority for modern surgeons.”

In plastic surgery, a review focused on traditional mentorship and supervising of residents in the OR (26). The authors concluded the traditional method of medical learning “see one, do one, teach one” was still applicable but extended the method by stating: “…in this era of outcome and evidence-based medicine, medical learning should be to see many, learn from the outcome, do many with supervision and learn from the outcome, and finally teach many with supervision and learn from the outcome” and continued: “However, the method can be built upon various learning principles, committed mentors, and advanced technology such as medical simulators. Residents must be encouraged and given the opportunities to learn and improve upon their surgical skills by mentors who continue to improve upon their skills as well. This can all be done in an environment that keeps patient safety at the forefront.”

A 2014 report from the Direction of Education of the American College of Surgeons (10), after a 3-month visit to Ireland where the Royal College of Surgeons in Ireland has long been actively developing surgical training methodologies (55), emphasized the importance of “remediation,” and discussed a methodology where the first corrective measure following an error was “to discuss over a cup of coffee,” and combined the assessment of technical and non-technical operative skills. One final conclusion was: “Trainee performance should be evaluated in a rigorous, reliable and meaningful way to ensure that graduates have the skills necessary for safe, independent practice” without defining how to do this.

A 2014 review on general surgery in Canada and the UK (11) examined evidence supporting the use of simulation in surgical education and concluded that “The effectiveness of surgical simulators has been demonstrated in randomized controlled trials. The evidence from those trials has been summarized in systematic reviews and meta-analysis. Simulation has also been used in selected high-stakes examinations. Unfortunately, despite all of this progress, simulation-enhanced training is still not a standard or a mandatory component of all surgical training. Evidence of improved clinical outcomes and cost-effectiveness of surgical simulation will be required to make simulation-enhanced education a mandatory component of surgical training, retraining, certification, and re-certification. With such evidence in hand, surgeon educators will finally have the leverage necessary to advocate for simulation-enhanced training with hospital administration, government officials, surgical credentialing bodies, and, most importantly, patients.”

Surgeon-specific outcome data (SSD) from different surgical specialties in the UK (56) was first reported in 2014, where it was pointed out that “…disregarding the lack of governmental instructions on how to perform surgical training and assessment this is regarded as a significant advancement in health service transparency.” Factors influencing statistics such as outcomes of life-saving procedures with poor initial prognosis were not specifically discussed.

An evaluation of the effectiveness of different forms of e-learning in 2016 (39), stated that: “Despite significant heterogeneity amongst platforms, e-learning is at least as effective as other methods of training,” having included 87 out of 4,704 articles meeting the quality requirements using the GRADE standards and the CONSORT checklist (Table 2) for RCTs to evaluate limitations in study quality and potential risk of bias. A total of 7,871 surgeons included in the 87 studies had been using some type of e-learning. Only two RCTs demonstrated a transfer of skill to the clinical environment, and there was no evidence to show improvement in patient outcomes. They also discussed the importance of using e-learning within a larger context, combining factors as follows: “focused feedback, immediate and ample opportunities for repetition, focused practice, and emphasis on difficult aspects and areas of weakness.”

An interdisciplinary group of experts in (1) computer engineering, (2) informatics, (3) biomedical, industrial and human factors engineering, and (4) surgery (57) evaluated factors of distraction in the operating room 2016 and concluded that: “Operating room protocols should ensure that distractions from intermittent auditory and mental distractions are significantly reduced. In addition, surgical residents would benefit from training for intermittent auditory and mental distractions in order to develop automaticity and high-skills performance during distractions, particularly during more difficult surgical tasks. It is unclear as to whether training should be done in the presence of distractions or distractions should only be used for post-training testing of levels of automaticity.” Assessment of “surgical performance” was measured in different ways in the included studies, e.g., time to completion of a task, number of errors without defining “error,” and increase in movements (influencing the time to completion). They concluded that: “Due to the lack of consistency between performance measures, missing experimental or results data, or variance in experimental design, aggregated findings (i.e., a meta-analysis) could not be performed.”

A 2017 review on general surgery from an international perspective originating from Italy and Saudi Arabia (51) included aspects of patient safety during surgical training. Virtual surgical simulators were seen as the solution for both training and assessment, but “at the moment, we cannot recommend a single superior model for surgical training and assessment.”

A systematic review of general surgery and minimally invasive surgery in Canada 2017 (58), focused on perioperative teaching and feedback, searching the terms: “teaching, feedback, guidance, or debriefing” in the perioperative period and concluded that: “There is a need for improved quality and quantity of structured feedback.” Four of the 26 included studies reported examples of deliberate implementation of teaching or feedback strategies in the OR. One conclusion was: “In subsequent cases, the feedback group demonstrated a significant improvement in time to complete the procedure, errors, and economy of movement compared to the group that did not receive feedback.”

A systematic review of surgical training on reperfused human cadavers (40) was presented from Belgium in 2018 where different techniques of reperfusion and ways of embalming were used in the included studies, and the authors concluded that: “Various reperfused human cadaver models exist, enabling practice of mainly vascular procedures. Preservation method determines the level of simulation fidelity. Thorough evaluation of these models as surgical training tools and transfer effectiveness is still lacking.”

A review of surgical simulation training in sub-Saharan Africa (SSA) in 2018 (59) reported: “An estimated 5 billion people worldwide lack access to any surgical care, whilst surgical conditions account for 11%–30% of the global burden of disease. Maximizing the effectiveness of surgical training is imperative to improve access to safe and essential surgical care on a global scale.”…and continued: “Few studies reported any outcome data. Compared to the volume of surgical training initiatives that are known to take place in SSA, there is very limited good quality published evidence for the use of simulation training in this context.”

In pediatric surgery (60), it was reported in 2019 that simulation courses increased proficiency in technical skills and were useful in preparing general surgeons for work in emergency situations. However, the exposure to real-life operative trauma management was judged as a remaining issue. It was concluded that: “Literature suggests that the quality of surgical care correlates with surgical experience” while the term “experience” was not defined.

A 2019 review on orthopedics, trauma, hand, and reconstructive surgery in Germany (16) including international aspects of evidence in surgical training stated that surgical curricula contained major differences even between the countries within the European Union. No studies analyzing the educational programs in different countries were found, and there is no evidence indicating which educational system is superior. The topic *assessment* was pinpointed: “There is also little evidence to distinguish the good from the average surgeon or the junior surgeons’ progress, during his residency training. Although some evaluation tools are already available, the lack of resources of most teaching hospitals often results in not using these tools as long it is not mandatory by a governmental program” and continued: “Most countries provide a curriculum for surgical education, but the programs differ in their structure and time points of assessment. For example, the United Kingdom and the Republic of Ireland use structured educational programs with repeating assessments for human factors, technical skills and medical knowledge using multiple-choice questionnaires—but practical tests are not performed, so the surgical procedural quality is not part of the certification.” It was expressed that no comparability studies between residents of different hospitals were performed. The authors advocated the use of validated scores such as the Ottawa Surgical Competency Operating Room Evaluation (O-SCORE), the Operative Rating System (OPRS), or the Zwisch scale (Table 2). In addition, it was stated that few published studies focused on the simulation of open surgery and that some studies showed: “Bench model training skills are transferable to the human model (cadaveric), but that yet no study analysed the further transfer of manual skills to the operating room.”

In a 2019 study on orthopedic surgery in the UK (32), the current status of cadaveric simulation for surgical training was analyzed, concluding: “There is an abundance of relatively low-quality evidence showing that cadaveric simulation induces short-term skill acquisition as measured by objective means. There is currently a lack of evidence of skill retention, and of transfer of skills following training into the live operating theatre.”

The incorporation of surgical training in international healthcare missions and its effect on the global burden of surgical disease was reported from general surgery in the USA in 2019 (61) by a systematic review. In an “ethical checklist” with 16 organizational factors, one question on “knowledge/skill translation” was included, coming from the field of neurosurgery. It was stated that: “The impact of international missions on the professional development of surgeons in training cannot be understated” and that “several studies have presented …improved surgical skills,” but the question about how such skills were measured was not discussed. The importance of “case volumes” was mentioned, not taking any question of surgical outcome or quality into account.

The terms simulation, VR, XR, augmented reality, and mixed reality as well as haptic techniques and computing graphics were explored in ENT surgery in 2020 (18). It was stated that: “High-fidelity simulation centres require a significant upfront investment, and that the operation of these centres requires an extensive team of expert staff and incurs large running costs. Cadaveric simulation has extremely high running costs and ethical implications using human cadavers. Both of these educational tools require the learner to attend a course on a specific date and generally run through the scenario just once. These courses or educational activities, to be maximally effective, may need to be repeated, thus incurring repeated similar costs.” VR training was mentioned to have an advantage of “repeatability, reliability and online access compared to such labs and to cadaveric dissection and temporal bone laboratories for ENT surgery, and that a virtual reality simulator is relatively inexpensive, requires little space and, depending on the educational model, no supervision.”

From medical and dental training centers in Northern Ireland (62), it was concluded in 2020 that: “Procedure-Based Assessment (PBA)” and “Direct Observation of Practical Skills (DOPS) assessments are educationally useful tools in postgraduate surgical training. Further research is required to determine the number of assessments required to ensure adequate reliability.”

In a 2020 study on trauma surgery in Pakistan (20), it was concluded that: “Current training in most surgical residency programs, locally and globally, is suboptimal” and “The decrease in volume of trauma cases results in variable and/ or inadequate exposure and hands-on experience of the surgical trainees in operative management of trauma” and stated that: “We have identified the deficiencies in the trauma-related training and education in postgraduate surgical education.” A set of components was recommended in the trauma surgery curriculum such as “video review with debriefing,” “simulated trauma resuscitation,” “computer-based programs and games,” and “simulation-based trauma scenarios of increasing difficulty such as surgery for abdomino-thoracic violence (SAVE),” and it was furthermore stated that: “The estimated increase in the burden of the trauma-related injuries in coming years warrants a multifactorial concerted effort to equip our surgeons with the essentials of knowledge and skills required to adequately manage trauma cases thus reducing resultant mortality and morbidity.” No suggestions of systematic or scheduled use of simulation systems or stepwise examinations of surgical skills were described in the review.

A 2020 review on the use of augmented reality (AR) training, covering the full range of surgical disciplines in the UK (63), reported the outcomes to be measured in many different ways, such as “Competency, operational/procedural duration, user opinion recorded through a Likert scale questionnaire (Table 2) and postoperative complication rate.” Only two of the included studies were set in a real clinical environment. AR training increased the scores for *competency, surgical opinion, and postoperative complication rate* compared with traditional training, while operative duration increased using AR. The term population, intervention, comparison, and outcome (PICO) was also used to report outcomes. It was concluded that: “Due to the lack of any common protocol, there was an almost infinite variability in trial design.” No suggestion of a systematic use of AR in surgical training programs was made.

A systematic review on surgical training on donated cadavers in the UK (41) stated that: “Out of 51 studies, 22 assessed the impact of the cadaveric training intervention using only subjective measures, 5 measured impact by change in learner knowledge, and 23 used objective tools to assess change in learner behaviour after training.” Only one study assessed patient outcomes and demonstrated the *transfer* of skill from the simulated environment to the workplace. Of the included studies, 67% had weak methodology (MERSQI score <10.7). It was concluded that: “There is an abundance of relatively low-quality evidence showing that cadaveric simulation induces short-term skill acquisition as measured by objective means. There is currently a lack of evidence of skill retention, and of transfer of skills following training into the live operating theatre.”

In a systematic review from Germany and Greece (42), 18 RCTs focusing on the very *transfer* of skills from simulation-based training in different laparoscopic procedures to skills in the operating room 2020 it was concluded that: “*Simulation provides a safe, effective, and ethical way for residents to acquire surgical skills before entering the operating room*.”

The terms “work-based assessments (WBA), procedure-based assessment (PBA), clinical evaluation exercise, formative assessment, case-based discussion, and direct observation of procedural skills” were analyzed in a 2021 review of general surgery in the UK (49), where it was concluded that: “In Miller's pyramid (Table 2), a framework for assessing clinical skills and performance, WBAs are ranked at the highest level for assessing doctors in action.”

In a profound review from Canada in 2021 (28), a comparison was made between VR training and traditional training, concluding: “Trainees demonstrated improvement of surgical skills through VR simulation, especially in comparison to non-VR, traditional training. Immersive VR trainees demonstrated faster procedural completion times, greater task completion scores, and improved accuracy of surgical device implantation when compared to control in multiple RCTs.” Their conclusion was: “Further work should be done to compare the efficacy of VR with lower fidelity simulators, and clarify its role in the context of large-scale, comprehensive surgical residency programs. This nascent technology has the potential to remodel our contemporary views of surgical training and serves as a compelling supplement to cadaveric and real patient training.”

A critical evaluation of key issues in laparoscopic general surgical training and digital technology and surgical skills assessment was made in the UK and Ireland in 2021 (64), concluding a more streamlined and efficient training system supporting cognitive, technical, and non-technical skills can be achieved due to the technical development. Standardizing and automating assessments were supposed to improve surgical outcomes. Digital strategies appear to benefit surgeons, surgical teams, and patients alike. Asking for more available data, it was suggested it could lead to standardized, regulated systems that can support surgical teams in providing safer surgical care.

Innovations in urological surgical training for the 5 last years were summarized in the USA in 2021 (65), and the structured surgical video review showed improved surgical skills not only for trainees but also for qualified surgeons. Despite the advances, it was concluded that there are still unfulfilled needs for a standardized surgical training program covering both technical and non-technical skills.

A scoping review from Gynecology in Denmark 2021 (66) searched for standardized assessment tools for gynecology surgical training, as consensus was considered to be missing on which surgical assessment tool to use. Assessment methods from 544 articles were categorized, and eight tools were identified for measuring technical skills during gynecologic surgery; however, all of these depended on the user context, with varying validity frameworks. Their conclusions were: “There is a need for larger, randomized studies to evaluate their validity before they are enrolled in gynaecological curricula or used for summative assessment” and that “the review can serve as a guide for surgical educators who want to apply a scale or a specific tool in surgical assessment.”

Concerning ophthalmologic robot surgery in Canada and the Netherlands (67), the role of industrial partners in surgical training and assessment was mentioned in 2021: “There is a lag in the development of a comprehensive training and credentialing system for robotic eye surgery, and certification of robotic skills proficiency relies heavily on industry leadership.” In addition, the idea of stepwise structured learning was recommended “in a stepwise, competency-based manner from didactic learning, to simulation exercises, to finally operative experiences,” and further: “We have developed an assessment tool based on validated global rating scales for surgical skills that may be used to monitor the progress of trainees. Finally, we propose a graduating model for granting privileges to robotic surgeons.”

A group of plastic surgeons in Italy, Germany, and the USA concluded in 2022 (44): “To date, there are no standardized or ideal simulation models for local skin flap teaching,” and “most of the described models have been assessed only in small cohort numbers, and therefore larger candidate sizes and standardized methods for assessment are required.”

Simulation-based training in cardiac surgery was reviewed by colleagues in the UK and the Netherlands in 2023 (30), and they stated that: “The results of the included studies suggest that validity assessment is scarce within the field, being carried out for only 4 of the models. Nonetheless, all studies reported improvement in trainees’ confidence, clinical knowledge and surgical skills (including accuracy, speed, dexterity) of trainees both at senior and junior levels. The direct clinical impact included initiation of minimally invasive programmes and improved board exam pass rates, and creating positive behavioural changes to minimize further cardiovascular risk” meaning that reasoning about “patient safety” was connected to the question of training methods.

In a major international review on surgical simulation in 2023 (68), the question about treating the subject of surgical training strictly scientifically, as any other method in healthcare, stated that: “Few previous studies compared learners who received structured simulation training to a group of trainees who did not receive any simulation training in single-centre randomized control research.” The use of videos was emphasized, the cost of setting up a simulation lab was estimated to range from $100,000 to $300,000, and it was stated that: “a good method of simulation training is using virtual reality simulators with haptics and simulated patients. The availability of these facilities is limited, though, and a typical session might include an exercise involving stacking sugar cubes and box trainers. The degree of expertise or competency is one area that needs clarification as medical education transitions to a competency-based paradigm.” [The last conclusion about the need for competency-based training was also the general conclusion of the First Surgicon Congress in 2011 (48)].

From the Netherlands, the use of different digital tools in surgical training in different surgical specialties was reviewed (31) in relation to dry and wet labs, including digital box trainers, (immersive) virtual reality (VR) trainers, robot surgery trainers, coaching and feedback, serious games, and the role of such tools in complete surgical curricula was discussed. The conclusion stated that: “While the efficacy of digital tools in enhancing technical surgical skills is evident—especially for VR-trainers—there is a lack of evidence regarding non-technical skills, and need to improve methodological robustness of research on new (digital) tools before they are implemented in curricula.”

In an extensive but highly specialized international review (69) on the use of Artificial Intelligence in the surgical training and treatment of kidney cancer from Italy, Egypt, Belgium, Portugal, Spain, Russia, Germany, the USA, and the UK, the possibilities of international collaboration were clearly demonstrated. They concluded that: “Potential AI applications in kidney cancer surgical training include analysing surgical workflow, annotating instruments, identifying tissues, and 3D reconstruction. AI is capable of appraising surgical skills, including the identification of procedural steps and instrument tracking. While AI and augmented reality (AR) enhance training, challenges persist in real-time tracking and registration. The utilization of AI-driven 3D reconstruction proves beneficial for intraoperative guidance and preoperative preparation. Artificial intelligence (AI) shows potential for advancing surgical training by providing unbiased evaluations, personalized feedback, and enhanced learning processes. Yet challenges such as consistent metric measurement, ethical concerns, and data privacy must be addressed. The integration of AI into kidney cancer surgical training offers solutions to training difficulties and a boost to surgical education. However, to fully harness its potential, additional studies are imperative.”

## Discussion

### General aspects

We searched the literature for generally applicable evidence-based methods for surgical training and assessment. That is, scientifically validated, internationally accepted methods for training and assessment of surgical skills, in review articles. Few attempts to harmonize surgical training methods and competence levels internationally were found. Despite all efforts found, paid by different working groups and institutions, the suggested solutions seem to be scattered and inconsistent. However, the resemblance of the conclusions in the included reviews was striking, generally stating that “data is missing” and “more research is needed.” The formal responsibility for technical surgical training and certification, credentialing, and privileging is shown to be randomly divided between local, regional, or national interest groups and surgical societies. In each country, each surgical specialty is thus bearing the burden of creating applicable methods for training and assessment, a sustainable training structure, and the transfer of technical surgical skills from models into the OR. As a consequence, surgical skills are not comparable within countries and even less between countries, with one exception referring to a review dealing with artificial intelligence used in surgery for kidney cancer.

### Decision-making in a divided medical profession

Viewing the need for structured and validated surgical skills training, this can be seen as a part of continuous medical education (CME). Decision-makers might come from non-surgical specialties, explaining why surgical topics are not often specifically addressed. Medical doctors might be divided into two separate groups: one group that treats disease or injuries by medication and another group that treats disease or injuries by manual work. When discussing CME, this distinction is seldom made, not taking the need for continuous surgical skills training into account separately. This view of two principally different groups of medical doctors could also be applied to nurses and other medical staff. Our views on manual training are thus also applicable to all kinds of staff performing manual medical procedures, which extrapolates the practical training issue to an even higher degree.

New surgical methods and the use of new surgical equipment could also be regarded as any new medical treatment and compared with the introduction of new pharmaceuticals. The surgical methods should thus be equally subjected to high-quality evaluation in prospective, randomized, and controlled (Phase 1, 2, and 3) studies collecting reliable data before new technical devices and new surgical methods are accepted for general use.

### Future research leadership and external observers

As more data are needed, a new form of a combined competency might be useful, such as a new title, e.g., “University professor in surgical teaching/learning,” combining knowledge in surgery and learning sciences, to develop a high-quality scientific research path in this field. The majority of authors of the included reviews and underlying articles were surgeons themselves, originally trained in different ways and who in addition might be critically regarded as scientifically biased if the studies were performed in their own surgical department or hospital. The topic could benefit from applying normal and strictly scientific principles such as RCTs, using external unbiased observers to evaluate results. Broader views and interdisciplinary studies might also be valuable, integrating data from the medical, educational, technical, financial, and ethical domains, such as human factors adopting new technical equipment and adapting technical equipment to human (surgeons) needs.

Likewise, the introduction of a mandatory “head training officer” at each surgical department might also be helpful, including scheduled working time dedicated to the leadership for practical manual training activities. Logically, surgeons should have a defined amount of their working time dedicated to manual skills training, due to our opinion.

### Future training methods and documentation

Training by simulation was generally found to be valuable by increasing manual skills in a broad sense. However, the term simulation was used to refer to many different levels of surgery-like situations, from suturing in fabrics to high-fidelity computerized simulation equipment for selected elective surgical procedures. Most of the studies dealt with endoscopic surgical procedures and coupled computerized simulation. Studies of training and assessment of skills in open surgery seem to present special challenges, as the simulation of open surgery is technically demanding most often meaning resource-intensive training by dissection or training on animals under general anesthesia or on donated human cadavers. In the not-too-distant future, video documentation of surgical procedures might replace traditional written descriptions in medical records. This might be required from health care authorities, insurance companies, and the patients themselves. The AI technology will soon be able to interpret and score surgical videos. Some Swedish orthopedic surgeons already give a CD to the patients when they are discharged from the hospital with a video of their actual ACL replacement surgery.

### Structure, assessment, and transfer

The *structure* of the training should not be neglected, meaning that skills should be acquired stepwise, and *stepwise assessed*, suggesting any form of practical examinations until we have evidence-based methods for assessment. In Figure 1, each level is marked with a white line, representing the practical examination of the achieved skills. Regarding assessment methods in the included reviews, they showed to be even less well-defined compared with training methods, and the term “competence” was sometimes used without any definition. Only a few reviews mentioned the term *transfer* of manual skills, meaning the use of acquired skills on models transferred to the real performance in the OR.

### Costs

Time and resources dedicated to surgical training and assessment might be hard to find in financially strained healthcare. To motivate the expenses, cost–benefit analyses could be used comparing costs for training with costs for surgical errors and complications and their long-term effects, such as prolonged morbidity, prolonged sick leave, reduced or impaired working capacity, or death. We believe such data are mostly unavailable, and a future way to collect such data is an international understanding of the importance of systematically and objectively registering avoidable surgical complications using the International Code of Diagnoses 10 (ICD 10) Codes T 81.0–T81.9. This will need a global paradigm shift within the surgical community by using the mentality of “no personal blame.” Surgeons are humans, and we all make errors at times. By avoidable errors, we mean that high-risk surgical efforts in life-saving procedures in high-risk patients should be excluded from such statistics.

### Surgeons and the surgical industry

A final but core factor was mentioned in one of the included reviews: the educational role of industrial suppliers of surgical equipment. Surgeons and the surgical industry are forever intertwined, and training and certification are sometimes arranged by industrial partners; however, each supplier is only offering training on their own products. As far as we know, there are no defined crossing lines within the operating room where the responsibility for the use of technical equipment is held by the hospital direction—or by the certifying supplier. We argue that the different roles of industrial suppliers and hospitals, in introducing new surgical equipment, should be defined by clear and simple rules.

## Conclusions

We tried to analyze the state of the art in surgical training and assessment, by searching review articles on scientifically validated, internationally accepted, and generally applicable methods. Our overall conclusion was that such general and validated methods still are missing and that most authors with few exceptions did not suggest any generally applicable solutions, but concluded that there is a profound need for further research on this topic. Most of the reviews were limited to one surgical procedure, one surgical specialty, or one geographical area. Manual training outside the operating room seemed generally to increase surgical skills in the OR.

To move forward from this kind of standstill, we take the permission to suggest the surgical staircase model to be used as a general basic template for the creation of new hypotheses for further prospective scientific research. More studies performed using the same template would allow a more uniform collection of data and distantly more uniform and internationally comparable surgical skills.

Defining the steps of increasing skill difficulty seems like an easy task, if a common understanding within each surgical specialty could be worked out. As an example in orthopedic surgery, minor hand and foot procedures and hip fracture surgery might be included in Step 1—or whatever collaborating orthopedic societies might choose. Practical examinations should be mandatory to allow the proceeding from Step 1 to Step 2, which might speed up the process and produce mature surgeons in less time—as another hypothesis that also needs to be confirmed or rejected. This kind of system might slowly be accepted even internationally for each specialty, collaborating “across borders and boards.” In some cases, all given steps would not be necessary to complete, for example, a specialized orthopedic spine surgeon might just need competencies A, B, C, F, and H.

Future scientific research in this field would also benefit from combining surgical competency with competencies from teaching and learning sciences. A separation of the topic could then be useful, into *training methods*, *training structures*, *assessment methods*, and *transfer of skills* from the training situation to the operating room.

A distant common goal is thus to create (1) international standards for each surgical specialty, including (2) increasing steps of difficulty and (3) stepwise examinations using available assessment methods. (4) External unbiased observers might be useful in evaluating surgical results.

Costs for surgical skills training could be studied compared with costs for avoidable surgical errors and their long-term effects, to motivate the investment of dedicated working time to skills training. This would need a (5) systematical and objective registration of avoidable surgical complications using the International Code of Diagnoses 10 (ICD 10) Codes T 81.0–T81.9. In turn, this will need a higher understanding of the “no-blame” principle. (6) Finally, defining the roles of the educational responsibility between educating hospitals and the surgical device industry would be beneficial, by drawing medical legal liability borderlines for surgical skills training and certification.

To date, no administrative body would be able to create, inform, and control internationally equal surgical skills by regulations and certification schemes, even if validated data were available. Instead, we believe in an easily accessible collection of validated data and a continuous extraction of practical recommendations drawn from this database. Such work might be performed by any neutral, non-profit, and scientifically based non-governmental organization (NGO). The given recommendations would be free to follow for any surgical department, slowly creating a uniform way of surgical training and assessment in the surgical community, even internationally, over time.
